# Epidemiology of cutaneous adverse drug reactions 

**DOI:** 10.5414/ALX01508E

**Published:** 2017-08-04

**Authors:** M. Mockenhaupt

**Affiliations:** Dokumentationszentrum schwerer Hautreaktionen (dZh), Universitäts-Hautklinik Freiburg, Germany

**Keywords:** cutaneous adverse drug reactions, drug exanthema, Stevens-Johnson syndrome, toxic epidermal necrolysis, acute generalized exanthematous pustulosis, drug reaction with eosinophilia and systemic symptoms, epidemiology

## Abstract

Epidemiologic investigation of cutaneous adverse drug reactions (cADRs) is important in order to evaluate their impact on dermatology and health care in general as well as their burden on affected patients. Few epidemiologic studies have been performed on frequent non-life-threatening cADR, including reactions of both delayed and immediate hypersensitivity, such as maculopapular exanthema (MPE), fixed drug eruption, and urticaria. Concerning rare but life-threatening severe cutaneous adverse reactions, e.g., toxic epidermal necrolysis (TEN), Stevens-Johnson syndrome (SJS), acute generalized exanthematous pustulosis (AGEP), and drug reaction with eosinophilia and systemic symptoms (DRESS), several epidemiologic studies have been performed to date, some of which are still ongoing. Such studies enable the calculation of reliable incidence rates and demographic data, and also allow researchers to perform risk estimation for drugs. The spectrum of drugs causing cADR differs substantially when separating the various clinical conditions. Whereas antibiotics are by far the most frequent inducers of milder cADRs, like MPE, they have a much lower risk of inducing SJS/TEN, for which “high-risk” drugs are anti-infective sulfonamides, allopurinol, certain anti-epileptic drugs, nevirapine, and non-steroidal anti-inflammatory drugs (NSAIDs) of the oxicam-type. In contrast, AGEP is predominantly caused by the antibiotics pristinamycin and aminopenicillins, followed by quinolones, (hydroxy-)chloroquine, and sulfonamides. DRESS can be induced by a number of drugs known to cause SJS/TEN, such as certain antiepileptics and allopurinol, but also other medications (e.g., minocyclin).

German version published in Allergologie, Vol. 35, No. 3/2012, pp. 131-144

## Introduction 

According to the WHO (World Health Organization) definition, an adverse drug reaction (ADR) is “a response to a drug which is noxious and unintended, and which occurs at doses normally used in man for the prophylaxis, diagnosis, or therapy of disease, or for the modifications of physiological function” [36]. Up to 80% of ADRs are dose-dependent and predictable, while about 20% are independent of the administered dose and unpredictable. Frequently, the latter are immunologically mediated reactions, often called “drug allergy”, and involve either IgE or T cells. In contrast, non-immunologically mediated reactions are called “idiosyncratic reactions” [8]. As a majority of ADRs involve the skin, epidemiological studies were mainly carried out on the topic of cutaneous manifestations. However, these studies often summarize various cutaneous adverse drug reactions (cADRs) with different mechanisms of development and clinical pictures. In addition to trials on anaphylaxis of varying origin, systematic large-scale epidemiological studies were also carried out on severe cADRs. Thanks to these studies, reliable data on the incidence and demography became available. Only few studies have been published on the epidemiology of mild cADRs. 

## Clinical picture of cutaneous adverse drug reactions 

The majority of cADRs are neither severe nor life-threatening. Nevertheless, many patients with cADRs have to stay in hospital for a while, because at onset, severe and life-threatening reactions have to be assumed or cannot be excluded. 

### Non-life-threatening cutaneous adverse drug reactions 

Milder forms of cADRs include maculopapular rash, fixed drug eruption, morbilliform rash, urticaria, purpura, vasculitis, and a number of other manifestations. Many of these reactions, possibly with the exception of fixed drug eruptions, are not only induced by drugs, but can also be triggered by various infections [3]. For some of these reactions, infections seem to be the more probable cause, e.g., in urticaria or vasculitis, while other reactions always occur when infections and specific drugs are present at the same time; e.g., Epstein-Barr virus and aminopenicillins always induce maculopapular rash. Many physicians know the clinical picture of these frequent and less severe reactions; nevertheless, clinical diagnosis should be supported by a dermatologist and confirmed by skin biopsy. Sometimes it is difficult to find out whether or not a specific reaction was caused by drugs. Thus, a detailed patient history for drug use is crucial. Further allergic work-up can be carried out about 2 – 4 months after the skin reactions have healed. 

### Life-threatening cutaneous adverse drug reactions 

These forms of reaction are called severe cutaneous adverse reactions (SCAR). They include Stevens-Johnson syndrome (SJS) and toxic epidermal necrolysis (TEN), but also acute generalized exanthematous pustulosis (AGEP), and hypersensitivity syndrome, which recently has become known as drug reaction with eosinophilial and systemic symptoms (DRESS) [14]. A clinical consensus definition for skin reactions in the range of SJS/TEN was the prerequisite for epidemiological studies and systematic data analysis. Well-defined diagnostic scores were developed for AGEP and DRESS. 

**SJS/TEN**

**Figure 1.**
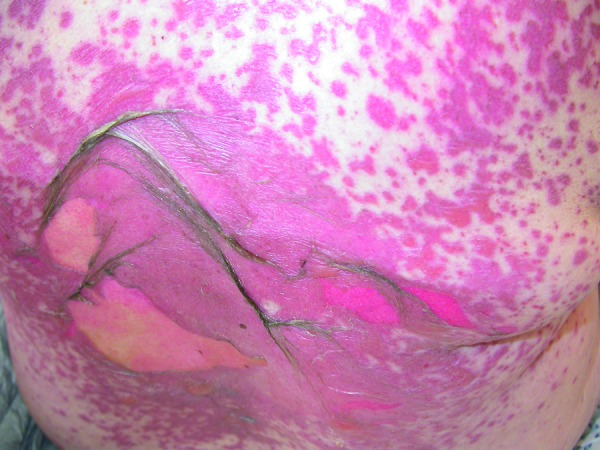
Spotted exanthema with skin detachment in SJS/TEN.

**Figure 2.**
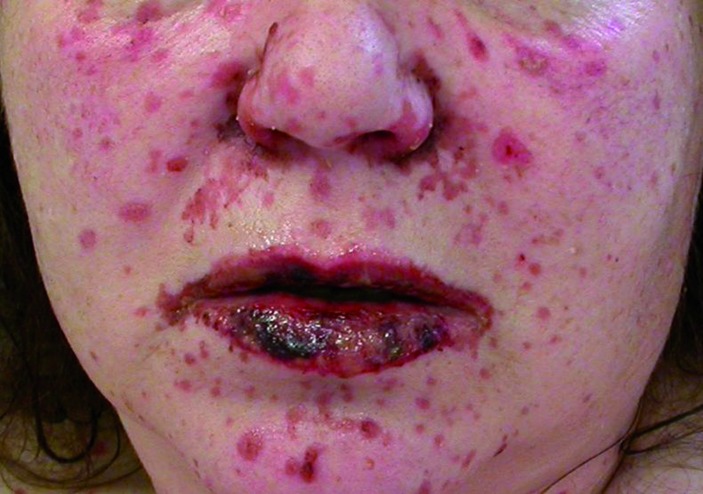
Hemorrhagic erosive lips and oral mucosa in SJS/TEN.

These reactions are characterized by erythematous skin eruptions and extensive epidermal detachment as well as mucosal erosions (Figure 1, Figure 2) [14]. SJS and TEN are considered to be different levels of severity of one and the same disease entity, which shares the same cause and underlying mechanism. The level of severity is characterized by the degree of epidermal ablation. In SJS, it is limited to less than 10% of body surface, while in TEN, epidermal ablation affects more than 30% of the body surface. If areas between 10% and 30% are affected, this is called an SJS/TEN intermediate form (Figure 1) [1, 14]. Histopathology shows a subepidermal clefting and necrotic keratinocytes, either in disseminated form or in as a complete epidermal necrosis. The underlying mechanism corresponds to an extensive apoptosis. Based on the almost identical histopathology of SJS/TEN and erythema exsudativum multiforme (EEM), SJS/TEN is frequently considered to belong to the broader spectrum of EEM [14]. For decades, EEM with mucosal involvement (EEM majus / EEMM) was considered to be the same as Stevens-Johnson syndrome, which led to an incorrect evaluation of its etiology. However, EEMM is mainly induced by infections and not by drugs [1]. A consensus definition allows the differentiation between SJS and EEMM according to the clinical picture [2, 29]. 

**AGEP**

**Figure 3.**
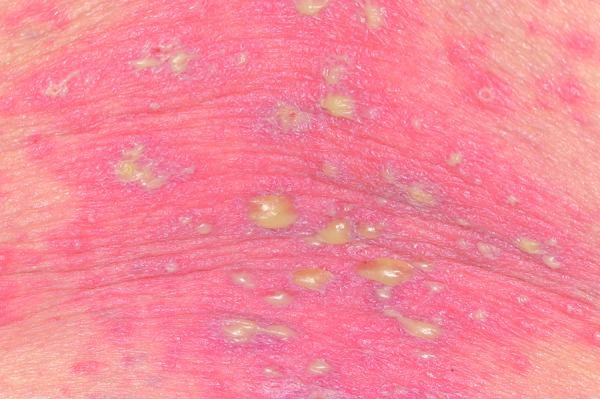
Non-folicular pustules in AGEP.

Acute generalized exanthematous pustulosis is characterized by the sudden occurrence of dozens of sterile, non-follicular, pinhead-sized pustules on an edematous erythema, which is mainly localized on the flexor surfaces (Figure 3). The reaction is frequently accompanied by fever and leukocytosis, particularly in the form of neutrophilia. The pustules develop within only a few hours and regress within a few days, leaving behind a typical post-pustule desquamation. Complications are rare, but can occur in patients in bad general condition [31]. Histopathology shows subcorneal and/or intraepidermal pustules, sometimes accompanied by a pronounced edema in the papillary dermis as well as perivascular infiltrates from neutrophils and eosinophils [31]. The occurrence of pustules frequently leads to the misdiagnosis of an infectious event so that an ADR is not suspected. 

**DRESS**

For many years, a high number of severe ADRs were summarized under the term hypersensitivity syndrome [34]. Now, one specific entity of hypersensitivity syndrome is distinguished from other ADRs and denominated “drug reaction with eosinophilia and systemic symptoms” (DRESS). While the term DRESS has been established mainly in Europe and North America, the term “drug-induced hypersensitivity syndrome (DIHS)” is used in Japan [9, 30]. DRESS/DIHS is characterized by highly variable skin eruptions, multi-organ involvement, lymphocyte activation (lymph node enlargement, lymphocytosis, atypical lymphocytes), eosinophilia, and frequently also by virus reactivation [9, 30]. The main characteristics, like skin lesions, fever, and organ involvement, can also be present in several types of infections, concomitant or underlying diseases. Therefore, it is essential to thoroughly analyze each symptom with regard to its possible relationship with the reaction. Not all signs and symptoms are always recognized early; in particular, asymptomatic findings, like eosinophilia and atypical lymphocytes, can be missed. Furthermore, visible skin lesions and increased specific laboratory values can occur at various time points during the course of DRESS/DIHS. 

### Epidemiological studies in cutaneous adverse drug reactions 

Studies on cADR, e.g., the Boston Collaborative Drug Surveillance Program, have provided important information on the type of reaction and the potentially causative drug, but were not designed to investigate the incidence and prevalence of the reactions, which could therefore only be roughly estimated. Recently, two prospective trials on the epidemiology of cADR in hospitals were carried out. The first (French) study analyzed cADRs after systemic intake or application of drugs in a specific hospital over a period of 6 months. All patients were examined by a dermatologist, and the drug use was evaluated by a pharmacologist. Based on 48 hospitalized patients in whom cADR was diagnosed, a prevalence of 3.6 per 1,000 hospitalized patients was calculated [5]. The second study was a prospective cohort study carried out in Mexico over a period of 10 months. This study demonstrated a prevalence of 7 per 1,000 hospitalized patients [6]. In South Korea, a mandatory electronic reporting system for immunologically mediated ADRs could identify 2,652 cases of ADR in a total of 55,432 hospitalizations over a period of 7 months. The study included cutaneous as well as non-cutaneous reactions. An allergist classified 532 reactions to be “significant drug hypersensitivity reactions”. 100 of these were new events, and 70% of them had a cutaneous manifestation. The overall incidence of ADRs was estimated to be 1.8 per 1,000 hospitalizations [21]. 

### Epidemiological studies in severe cutaneous adverse drug reactions 

In the past 25 years, several epidemiological studies on SCADR were carried out in Europe. In the 1980s, two hospital-based retrospective trials over a period of 5 years were carried out in France and Germany [24, 28]. In 1990, a prospective population-based registry for severe skin reactions was initiated in Germany. It aimed at the systematic recording of all hospitalized cases of SJS and TEN in Germany [26]. In parallel, an international case-control study on severe cutaneous adverse reactions (the so-called SCAR study) was carried out in Germany, France, Italy, and Portugal between 1989 and 1995 [12, 25]. Finally, a European case-control study on severe cutaneous adverse reactions (so-called EuroSCAR study) was carried out in Germany, France, Israel, Italy, the Netherlands, and Austria between 1997 and 2001 [15]. The EuroSCAR study examined SJS, TEN, and AGEP [32]. In 2003, the European registry on SCAR to drugs and collection of biological samples (also called RegiSCAR) was initiated to systematically collect cases of SJS/TEN, AGEP, and DRESS. At first, the RegiSCAR project started in the same 6 countries as the EuroSCAR study, later further partners from Taiwan, Spain, South Africa, and the United Kingdom participated [20]. These epidemiological studies established clinical networks for the active collection of cases. 

In the *Dokumentationszentrum schwerer Hautreaktionen (*dZh), the German registry for the documentation of severe skin reactions, a population-based approach was chosen, and a network of approximately 1,700 hospitals, including all their departments of Dermatology, Pediatrics, Burn Units, and Internal Medicine that offer intensive care treatment was built. Potential cases are reported to the dZh by phone, fax, or e-mail. A physician checks the inclusion criteria and organizes a visit to the reporting or treating hospital if the criteria were fulfilled. To guarantee a high reporting rate, all participating departments receive a quarterly reminder via a previously determined contact person. The hospitals are asked to reply using a postage-paid reply postcard to report cases of SCADR that occurred during the past 3 – 4 months and have not yet been reported. A high percentage of postcards are sent back to the dZh, but departments are contacted by phone if they do not report cases and do not respond to the reminders for a certain period of time. Thanks to such active and systematic measures of documentation, it can be assumed that cases of SJS and TEN in Germany are comprehensively recorded [26]. 

The *SCAR and EuroSCAR studies included cases reported to the dZh, while the other participating countries first had to build up specific networks.* Their work was not nationwide and thus not population-based, but followed the same rules for the documentation of cases. Nevertheless, only prospectively documented and directly interviewed cases of SJS/TEN that developed before admission to hospital and led to hospitalization were included in the case-control studies [12, 15, 25, 32]. For case-control analysis, 3 control patients were analyzed who were matched to each patient according to age, gender, region, and date of interview. Control patients were patients hospitalized due to acute illness, including infections (e.g., pneumonia), traumas (e.g., fractures), and abdominal emergencies (e.g., appendicitis, ruptured ovarian cyst, trapped hernia). Patients only served as controls if these acute illnesses were not associated with an underlying chronic disease. Not only cases, but also controls, were examined with regard to their diagnosis and eligibility, and inadequate controls were excluded. 

The ongoing *RegiSCAR study also includes cases developing in hospital (~ *1/3 of all cases). It studies cases of SJS/TEN, AGEP, and DRESS. The dZh and EuroSCAR networks are also used for the RegiSCAR project. The RegiSCAR project follows a cohort of patients to investigate long-term complications and patients’ quality of life after the severe skin reaction. In addition, blood samples are drawn for pathophysiologic examination and stored in a central archive [20]. 

All patients with severe skin reaction included in the studies were/are interviewed by trained medical personnel (physician, pharmacist, specially trained nurse) using a standardized questionnaire. It contains questions on the currents disease, demographic data, recent or past patient history, recent infections as well as detailed information on drug use. All documented cases are evaluated by a dermatology expert panel (Prof. K. Bork, Mainz; Prof. K.-F. Haustein, Leipzig; PD Dr. D. Vieluf, Meißen) according to the consensus definition and with regard to the clinical picture using clinical data, photographs, and histopathology. The experts do not have any information on drug exposure and infections. The cases are classified as “certain”, “probable”, “possible”, or “no” severe skin reactions [2, 29]. 

### Incidence and demographic data 

**SJS/TEN**

Although the data for the retrospective trials were not primarily collected for epidemiological use, a TEN incidence rate of 1.2 per 1 million inhabitants per year for France and of 0.93 per 1 million inhabitants per year for Germany could be calculated [24, 28]. Other countries reported incidences for SJS and TEN between 1.4 and 6 per million individuals per years. The high variability of these incidence rates could be caused by smaller reference populations, different diagnostic criteria, or other methodological problems (e.g., use of automated databases with different coding for the identification of cases) [33]. 

For the past 20 years, the population-based documentation carried out by the documentation center for severe skin reactions could calculate an SJS/TEN incidence of 1 – 2 cases per 1 million inhabitants per year (variation between 1.53 and 1.89) [16, 26]. 

SJS and TEN occur in different age groups. An average age of 53.4 years (1 – 94 years) was calculated for more than 2,200 patients with validated SJS/TEN. 36% of SJS patients were ≤ 40 years old, while 75% of patients with SJS/TEN intermediate form and 72% of patients with TEN were > 40 years old. In contrast, 83% of patients with EEMM were ≤ 40 years in age. A more specific analysis showed that 45% of EEMM patients were children and adolescents ≤ 17 years, while only 13% of SJS patients were ≤ 17 years old. In less than 8%, a clear distinction between EEMM and SJS could not be made, but these cases showed the demographic characteristics of EEMM. Men are more frequently affected by EEMM (66%), while SJS/TEN is more frequent in women (57 – 63%) [14]. 

In Europe, about 5% of SJS/TEN patients are HIV-positive, with this percentage having decreased over the past years. Although the age and gender distribution is different from that of HIV-negative patients with severe skin reactions, the mortality and the course of the reactions are comparable [4, 14]. The fatality rate is 9% for SJS, 29% for SJS/TEN intermittent form, and 48% for TEN, corresponding to a total fatality rate of 25%. The mortality rate is higher than it had been in previous years, which is probably due to the increased age of the patients (in a generally ageing society) and reflects the patients’ underlying diseases. Frequently, it is difficult to find the real causes of death in SJS/TEN patients; therefore, death within 6 weeks of occurrence of the skin reaction is considered to have a direct relationship. Risk factors for death within the acute phase of the reaction include a high degree of skin detachment, high age, and underlying diseases (e.g., renal disease, hepatic dysfunction, acute cancer). The RegiSCAR cohort study could also demonstrate a higher death rate 1 year after the acute phase of the severe skin reaction [17]. 

**AGEP**

The EuroSCAR study included the largest validated cohort of AGEP patients. As this case-control study was not population-based, reliable incidence rates for AGEP are not available. However, AGEP seems to be more frequent in some European countries than in others, which may be due to the availability of specific drugs with a high AGEP risk. 

Of the 150 possible AGEP cases documented in the EuroSCAR study, 97 could be validated as “probable” or “certain”. The majority of patients (78/97) were reported from France. The average age was 56 years (4 – 91 years), and 80% of patients were female. A death rate of almost 4% was calculated [32]. 

**DRESS**

No reliable numbers on the incidence of DRESS are available due to the fact that various adverse reactions were for a long time summarized under the term hypersensitivity syndrome and no epidemiological studies have been carried out. Existing data describe an incidence between 1 : 1,000 and 1 : 10,000 of exposed patients for “anticonvulsant hypersensitivity syndrome” [34]. 

Of the 201 cases documented in the RegiSCAR project, 177 could be validated as “probable” or “certain”. Interestingly, the median age of the 66 female patients was 41.5 years, while for the 51 male patients it was 57 years and thus considerably higher. With 2 of 117 cases, the death rate was clearly lower than the previously estimated 10% [10]. 

### Etiology of cutaneous adverse drug reactions 

A high number of case reports and case series report innumerable drugs that are made responsible for various forms of cADRs. A drug or drug group can induce completely different clinical reactions. On the other hand, a specific adverse reaction can be caused by many different drugs. Some substances seem to have an increased risk for cADRs, particularly in cases of concomitant virus infections, as described above for aminopenicillins and Epstein-Barr virus. In the above-mentioned epidemiological study from France, beta-lactam antibiotics were made responsible for 21% of cADRs [5], while in a Korean study, antibiotics were considered to be the causative agent in 32% of cases, followed by contrast media in 26% of cases [21]. In Singapore, antimicrobial substances and antiepileptics were the most frequent inducing agents (75%), followed by penicillins (25%), cephalosporins (16%), cotrimoxazole (9%), phenytoin (8%), and carbamazepine (6%), while allopurinol was less frequently identified to be the underlying factor (5.7%). It is, however, important to note that these percentages refer to delayed-type as well as immediate-type (63% maculopapular rash, 18% urticaria) cADRs [35]. In a 20-year study of cADRs in Switzerland (Comprehensive Hospital Drug Monitoring / CHDM), penicillin was considered to be the causative agent in 8% of cases and cotrimoxazol in 2.8% of cases. In more than 90% of the reactions, maculopapular drug eruption was diagnosed, while 5.5% of the reactions were urticaria, 1.4% vasculitis, and 0.5% were fixed drug eruption [7]. 

### Etiology and drug-related risk of severe cutaneous adverse drug reactions 

#### SJS/TEN 

Case-control studies are considered the gold standard for drug-related risk evaluation. In the EuroSCAR study, the risk evaluations are based on 379 so-called “community-acquired” cases of SJS/TEN (i.e., patients in whom the reaction developed outside the hospital and who had to be hospitalized due to this reaction) and 1,505 controls, all with fixed index date and adequate information on drug exposure. The evaluation considered, in particular, drugs that had already been suspected to cause adverse reactions, drugs that had been authorized shortly prior, but also drugs with a known risk of SJS/TEN, and drugs in which the problem of confounding could be present. Confounding occurs when, for example, a drug is used for an indication that on its own could have caused the reaction, for instance a certain infection. It can also be a problem when a drug is prescribed to treat symptoms that already represent the onset of an adverse reaction, like fever or malaise. This happens relatively frequently for antipyretics or analgesics [12, 15, 25]. 

Among the newer substances, lamotrigine and nevirapine were strongly associated with SJS/TEN. The manufacturers of both drugs had claimed that adverse reactions could be avoided by slow up-titration, which is obviously not the case for the occurrence of SJS/TEN [15]. For many previously suspected drugs, like antibacterial sulfonamides (in particular co-trimoxazole), allopurinol, carbamazepine, phenytoin, phenobarbital, and non-steroidal anti-inflammatory drugs (NSAIDs), a high risk could be confirmed [15, 25]. 

**Figure 4.**
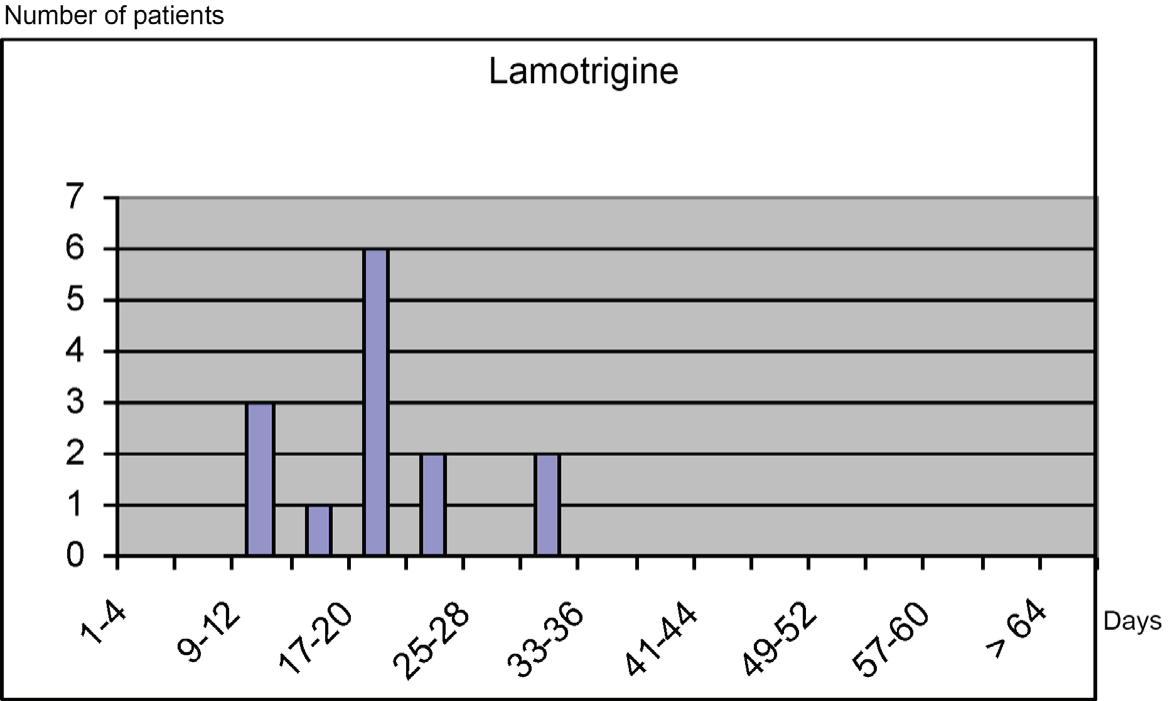
Latency between start of drug intake and onset of SJS/TEN for highly suspected / strongly associated drugs.

Most strongly suspected / associated drugs are prescribed and taken for longer periods of time. Almost all SJS/TEN patients who had been exposed to these substances (85% – 100%) had started intake less than 8 weeks before onset of the reaction (Table 1). The median latency time between start of drug intake and onset of the severe skin reaction was less than 4 weeks (15 days for carbamazepine, 24 days for phenytoin, 17 days for phenobarbital, 20 days for allopurinol) (Figure 4). The majority of patients with allopurinol exposure (56/66) had started intake shortly prior, in contrast to 1/27 controls. The univariate relative risk for recent use was 261 (36 – infinity), the multivariate relative risk for long-term use was 0.9 (0.3 – 2.4). After more than 8 weeks, no significant risk was observed, neither for allopurinol nor for other strongly associated drugs. The typical pattern for these drugs is characterized by a recent start of intake and missing or rarely taken concomitant medication with other highly suspected / strongly associated drugs (Figure 4, Table 1) [15, 25]. 

**Figure 5.**
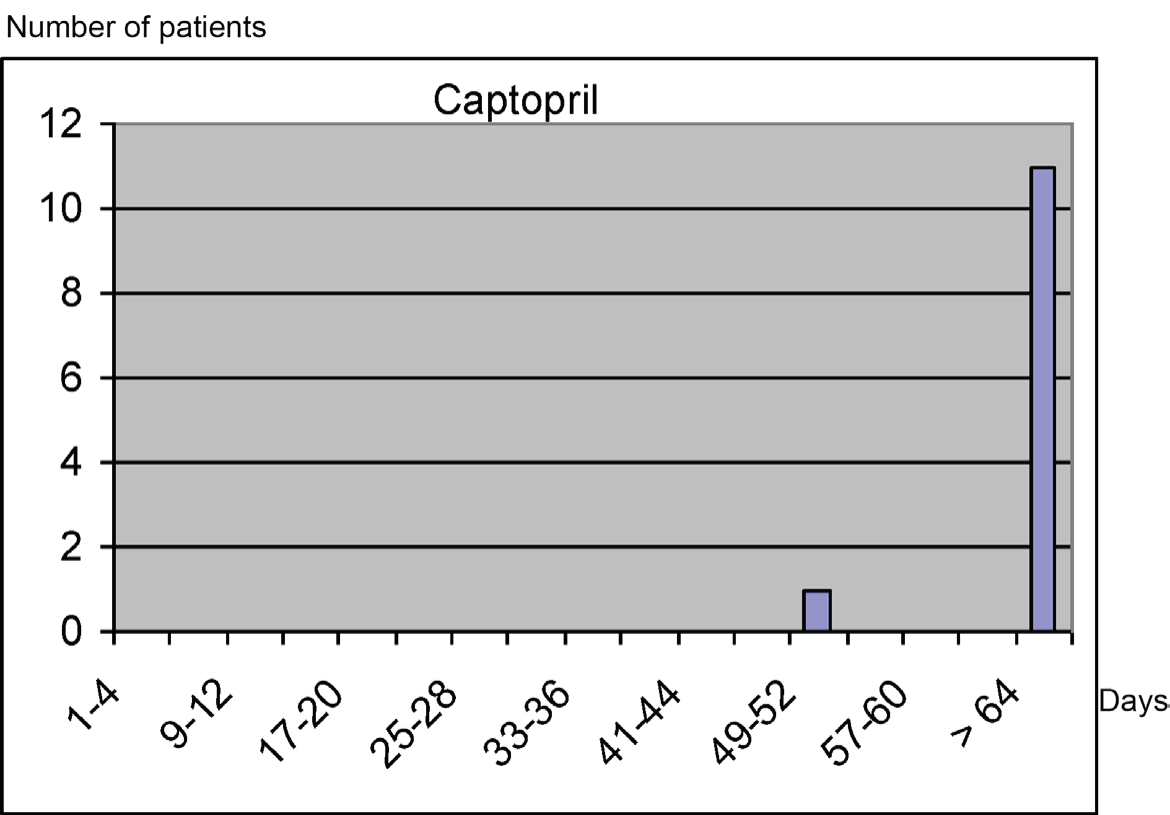
Latency between start of drug intake and onset of SJS/TEN for not suspected / not associated drugs.

For some other substances that were suspected to have an increased SJS risk, a significant but lower risk could be calculated. Among these suspected / associated drugs were various antibiotics (with penicillins having the lowest relative risk) and non-steroidal anti-inflammatory drugs (NSAIDs) of the acetic acid type, like diclofenac (Table 2). For valproic acid, it could be shown that, in contrast to previous assumptions, it does not pose an increased risk of developing SJS/TEN (uvRR = 9.4 (3.9 – 23), mvRR = 2.0 (0.6 – 7.4); latency of more than 30 weeks) [15, 25]. A high number of frequently used drugs and drug groups, like beta-blockers, ACE inhibitors, calcium antagonists, thiazide diuretics, furosemide, sulfonylurea antidiabetic drugs, insulin, and propionic acid NSAIDs (e.g., ibuprofen), were not associated with an increased SJS/TEN risk (Table 3, Figure 5) [15, 25]. 

Besides case-control studies, risk evaluations can be made by relating data on drug use in certain populations, e.g., SJS/TEN patients, with the numbers of prescriptions of certain drugs. For instance, the population-based dZh data were compared with the nationwide numbers of prescriptions, and the drug-related incidences were calculated for various NSAIDs. This analysis demonstrated lower risks for most NSAIDs, except for elonic acid derivatives [18]. Due to the fact that the risk for SJS/TEN is highest when the drug in question is taken for the first time, the percentage of first use among the prescriptions in Germany, the average prescribed dose as well as the duration of administration was estimated in the IMS Disease Analyzer-Mediplus database. This approach was based on all data of hospitalized patients with SJS/TEN who had been documented by the dZh and who had taken antiepileptic drugs. Analysis showed that more than 90% of patients developed the reaction within the first 63 days of drug use. The increased rates for prescriptions or dispensing over a 4-year period were 5% for carbamazepine, 65% for lamotrigine, 6% for phenobarbital, and 26% for valproic acid; for phenytoin the numbers decreased by 16%. The risk evaluations were carried out after various assumptions about the frequency of first use had been made; for various antiepileptic drugs (except valproic acid) a first-user rate between 1 per 10,000 and 10 per 10,000 was assumed [19]. 

Another option to estimate the risk in large cohorts of SJS/TEN patients is the application of standardized algorithms for causality evaluation. In 2010, the “algorithm for assessment of drug causality in Stevens-Johnson syndrome and toxic epidermal necrolysis (ALDEN)” was published. This algorithm provides structured help for the identification of the responsible drug(s) in an individual patient [27]. It includes the results of the above-described epidemiological studies and is based on the following criteria: latency between start of drug intake and index day (day of onset of the severe skin reaction), presence / availability of the drug in the body before index day (taking into account the drug’s half-life and the patient’s hepatic and renal function), information on previous and later intake as well as the discontinuation of the drug (if available), type of drug and its possible induction potential (based on drug lists that have to be updated regularly), and alternative reasons. Numeric score values allow the causality evaluation of every single drug a patient used 4 weeks before the reaction. The values are classified as “very improbable”, “improbable”, “possible”, “probable”, or “very probable” [27]. 

Nevertheless, drugs could be identified as a cause in only 75% of cases, i.e., in at least 25% of cases no causative drug could be detected, although SJS/TEN without drug is a very rare phenomenon. Some patients are on maintenance therapy that cannot be the cause of severe skin reactions. Viral infections and mycoplasma pneumonias have also been described to be etiologic factors, and a possible interaction of infections and drugs as well as an interaction between various drugs has not been elucidated yet. Frequently, it is difficult to decide whether certain symptoms, e.g., soreness of nasal and oral mucosa or conjunctival injection, have to be classified as signs of an acute infection or as the onset of a severe skin reaction. This can lead to difficulties in the identification of the index day and to an incorrect evaluation of causality. 

As no reliable in-vitro or in-vivo procedures are available that would allow researchers to evaluate the correlation between a specific drug and SJS/TEN in an individual case, the determination of the inducing drug is mainly based on the time interval between start of drug intake and onset of the reaction. For safety reasons, oral provocation testing with the suspected drug cannot be recommended, although the reaction does not necessarily re-occur at re-exposure, as shown by Finish studies from the 1970s. Patch testing with the potentially reaction-inducing drug are safe, but frequently result in false negatives [11, 16]. 

**AGEP**

Although case series frequently list drugs suspected to having caused AGEP, the first risk calculation was carried out in the EuroSCAR study using the case-control approach. Of the 97 patients with AGEP, 13 had taken macrolide antibiotics during the week before the index day. 10 of these 13 patients had taken pristinamycin, a substance registered in France. In 9 patients the reaction occurred only 1 day after intake, in 1 patient the reaction developed after 2 days. A high odds ratio was also calculated for aminopenicillins, with the exposure time being less than 15 days in all cases, and most patients used this substance only for a very short period of time. The results for quinolones were comparable. Seven cases and 2 controls were exposed to chloroquine or hydroxychloroquine, 7 cases and 10 controls to diltiazem (Table 4). None of these cases used concomitant medication that would carry a high risk of developing AGEP. Thus, it can be concluded that the anti-malaria drugs and diltiazem carry a high risk of developing AGEP, which could not be shown for other ACE inhibitors. The latency time between start of drug intake and onset of AGEP varied between a median of 1 day for antibiotics and suflonamides (41 cases in total) and a median of 11 days for other substances including chloroquine/hydroxychloroquine and diltiazem [32]. 

**DRESS**

So far, information on DRESS-inducing drugs has been based on case reports and case series. The reactions were frequently named after the drug that was considered to be causative, e.g., allopurinol hypersensitivity syndrome, dapsone syndrome, anticonvulsants syndrome, and so on. The RegiSCAR study analyzed the drug exposure in 117 validated cases of DRESS. A mean of 5 drugs (median; interquartile range 2 – 8) were taken in the month before the onset of the reaction. In 77% of the cases, 1 drug could be identified as having induced the reaction “probably” or “very probably”; in 3% of the cases, 2 drugs were identified. In 4% of the cases, the search for the causative agent remained unsolved, in 7%, the drugs taken were classified as “improbable”. Antiepileptics, including carbamazepione, phenytoin, and lamotrigine, were seen as causative agents in 36% of the cases, allopurinol in 18%, and sulfonamides in 12%. 

Other drugs were identified significantly less often. The mean latency between start of drug intake and onset of DRESS was 28 days (median; ± 17 days) for drugs with “probable” and “very probable” causality [9]. 

Furthermore, virus reactivation could play a major role in the development of DRESS, particularly when repeated events and persisting reactions occur [30]. However, it is not yet clear whether the reactivation of HHV6 or other members of the human herpes virus family play a causative role in the development of the reaction, or if the reactivation has to be interpreted as a complication [9, 23]. 

## “Genetic epidemiology” of cutaneous adverse drug reactions 

A genetic disposition to cADRs has long been assumed. Nevertheless, specific reaction types in relation to certain drugs could only recently be determined for patients with specific HLA patterns, which vary according to ethnicity. For example, HLA-A*3101 was shown to be present in European patients with carbamazepine-induced adverse reactions, particularly maculopapular rash, but not in severe reactions like SJS/TEN [13]. A very strong association between carbamazepine-induced SJS/TEN in Han-Chinese patients and HLA-B*1502 was observed which could not be confirmed in Europeans. HLA-B*5801 was found in Han-Chinese patients with SJS/TEN and DRESS after allupurinol intake (100%) as well as in Europeans with SJS/TEN (55%). These results are very important because they clearly show that first, the genetic predisposition for the development of SCADRs is highly associated with specific drugs, and that second, ethnicity plays a more important role than expected [22]. 

**Table 1.** Relative risk (RR) of induction of SJS/TEN for highly suspected / strongly associated drugs. Modified according to [15].

**Drug ** **Duration of intake**	**Patients ** **n = 379 (%)**	**Controls ** **n = 1,505 (%)**	**Univariate RR ** **(95%CI)**	**Multivariate RR ** **(95%CI)**	**Number of cases (%) with intake of “highly suspected” drugs within 8 weeks**
Nevirapine ≤ 8 weeks > 8 weeks	21 (5.5) 20 1	0 0 0	> 22 > 22 > 0.1	n.d.	0 (0%) 0 (0%) n.d.
Lamotrigine ≤ 8 weeks > 8 weeks	14 (3.7) 14 0	0 0 0	> 14 > 14 n.d.	n.d.	1 (7%) 1 (7%) 0
Cotrimoxazole ≤ 8 weeks > 8 weeks	24 (6.3) 19 5	1 (0.1) 0 1	102 (14 – 754) > 20 21 (2.3 – 172)	n.d.	4 (17%) 0 (0%) 4 (80%)
Other anti-infection sulfonamides ≤ 8 weeks > 8 weeks	13 (3.4) 13 0	1 (0.1) 1 0	53 (7.0 – 410) 53 (7.0 – 410) n.d.	n.d.	0 0 0
Allopurinol ≤ 8 weeks > 8 weeks	66 (17.4) 56 10	28 (1.9) 1 27	11 (7.0 – 18) 261 (36-∞) 1.4 (0.7 – 3.0)	18 (11 – 32) n.d. 0.9 (0.3 – 2.4)	7 (11%) 2 (4%) 5 (50%)
Carbamazepine ≤ 8 weeks > 8 weeks	31 (8.2) 29 2	4 (0.3) 0 4	33 (12 – 95) > 32 2.0 (0.4 – 121)	72 (23 – 225) n.d. n.d.	1 (3%) 0 (0%) n.d.
Phenytoin ≤ 8 weeks > 8 weeks	19 (5.0) 17 2	3 (0.2) 0 3	26 (7.8 – 90) > 17 2.7 (0.4 – 16)	17 (4.1 – 68) n.d. n.d.	3 (16%) 2 (12%) n.d.
Phenobarbital ≤ 8 weeks > 8 weeks	20 (5.3) 17 3	5 (0.3) 1 4	17 (6.2 – 45) 71 (9.4 – 532) 3 (0.7 – 13)	16 (5.0 – 50) n.d. 2.4 (0.2 – 23)	3 (15%) 2 (12%) 1 (33%)
Oxicam-NSAIDs^2 ^ ≤ 8 weeks > 8 weeks	11 (2.9) 11 0	7 (0.5) 3 4	6.4 (2.5 – 17) 15 (4.1 – 54) 0 (0 – 6.2)	16 (4.9 – 52) 50 (12 – 211) n.d.	1 (9%) 1 (9%) n.d.

^1^including sulfasalazine (5 cases, 1 control), sulfadiazine (5,0), sulfadoxine (2,0) sulfafurazol (2,0); ^2^including meloxicam (2,2), piroxicam (6,4), tenoxicam (3,1). n.d. = not done, due to < 3 cases or controls.

**Table 2.** Relative risk (RR) of induction of SJS/TEN for associated drugs. Modified according to [15].

**Drug ** **Duration of intake**	**Patients ** **n = 379 (%)**	**Controls ** **n = 1,505 (%)**	**Univariate RR ** **(95%CI)**	**Multivariate RR ** **(95%CI)**	**Number of cases (%) with intake of “highly suspected” drugs within 8 weeks**
Acidic acid NSAIDs3 ≤ 8 weeks > 8 weeks	27 (7.1) 24 3	21 (1.4) 11 10	5.4 (3.0 – 10) 9.2 (4.5 – 19) 1.2 (0.3 – 4.4)	5.6 (2.6 – 12) 13 (5.2 – 31) 0.7 (0.1 – 3.3)	7 (26%) 7 (29%) 0
Macrolides^4^	18 (4.8)	10 (0.7)	7.5 (3.4 – 16)	6.8 (2.6 – 18)	8 (44%)
Quinolones5	13 (3.4)	5 (0.3)	10.7 (3.8 – 30)	6.9 (1.8 – 27)	6 (46%)
Cephalosporines^6^	19 (5.0)	7 (0.5)	11.3 (4.7 – 27)	7.3 (2.4 – 22)	12 (63%)
Tetracyclines^7^	7 (1.9)	5 (0.3)	5.6 (1.8 – 18)	6.3 (1.6 – 25)	1 (14%)
Aminopenicillins^8^	18 (4.8)	18 (1.2)	4.1 (2.1 – 8.0)	2.4 (1.0 – 5.9)	10 (56%)

^3^including diclofenac (21,17), indomethacin (1,2), lonazolac (2,0), etodolac (1,1), aceclofenac (1,0), sulindac (1,0), ketorolac (0,1); ^4^including azithromycin (3,1), clarithromycin (4,5), erythromycin (3,0), midecamycin (0,1), pristinamycin (1,0), roxithromycin (4,2), spiramycin (3,1); ^5^including ciprofloxacin (6,2), grepafloxacin (1,0), levofloxacin (1,0), norfloxacin (4,2), ofloxacin (1,1); ^6^including cefaclor (0,2), cefalexin (3,0), cefapirin (1,0), cefatrizine (2,0), cefixime (3,2), cefonicide (1,2), cefotiam (2,0), cefpodoxim (0,2), ceftibutem (0,1), ceftriaxon (3,0), cefuroxim (5,0); ^7^including doxycycline (3,5), metacycline (1,0), minocycline (3,0); ^8^including amoxicillin (17,18), bacampicillin (1,0).

**Table 3.** Relative risk (RR) of induction of SJS/TEN for non-associated drugs. Modified according to [15].

**Drug ** **Duration of intake**	**Patients ** **n = 379 (%)**	**Controls ** **n = 1,505 (%)**	**Univariate RR ** **(95%CI)**	**Multivariate RR ** **(95%CI)**	**Number of cases (%) with intake of “highly suspected” drugs within 8 weeks**
Beta-blockers	37 (9.8)	122 (8.1)	1.2 (0.8 – 1.8)	0.9 (0.5 – 1.5)	19 (51%)
ACE inhibitors	44 (11.6)	120 (8.0)	1.5 (1.1 – 2.2)	0.9 (0.5 – 1.5)	23 (52%)
Calcium antagonists	45 (11.9)	104 (6.9)	1.8 (1.3 – 2.6)	1.4 (0.8 – 2.4)	24 (54%)
Thiazide diuretics	26 (6.9)	80 (5.3)	1.3 (0.8 – 2.1)	0.7 (0.4 – 1.4)	17 (65%)
Furosemide	41 (10.8)	49 (3.3)	3.6 (2.3 – 5.5)	1.8 (0.9 – 3.4)	24 (59%)
Propionic acid NSAIDs	16 (4.2)	35 (2.3)	1.9 (1.0 – 3.4)	1.5 (0.6 – 3.4)	8 (50%)
Sulfonylurea antidiabetics	11 (2.9)	35 (2.3)	1.3 (0.6 – 2.5)	0.8 (0.3 – 2.4)	5 (45%)
Insulin	10 (2.6)	22 (1.5)	1.8 (0.9 – 3.9)	1.0 (0.3 – 3.3)	6 (60%)

**Table 4.** Relative risk (indicated as OR) of induction of AGEP for strongly associated drugs. Modified according to [32].

**Drug or drug group**	Patients **n = 97 (%)**	**Controls ** **n = 1,009 (%)**	Odds ratio*	(95%CI) **		Number of cases (%) with intake of other “highly suspected” drugs within 8 weeks***
Pristinamycin	10 (10)	0	∞	26	∞	1 (10%)
Aminopenicillins	18 (19)	17 (2)	23	10	54	3 (17%)
Quinolones	9 (9)	5 (0.5)	33	8.5	127	3 (33%)
(Hydroxy-) chloroquins	7 (7)	2 (0.2)	39	8.0	191	0
Antibacterial sulfonamides	4 (4)	0	∞	7.1	∞	0
Terbinafin	4 (4)	0	∞	7.1	∞	1 (25%)
Diltiazem	7 (7)	10 (1)	15	5.0	48	0

>*multivariate odds ratio if at least 3 cases and 3 controls were exposed, otherwise univariate odds ratio; **CI = confidence interval; ***exposure to highly suspected drugs = the other substances listed in the table.
